# Diseases of Economic Importance in Feedlot Cattle in Sub-Saharan Africa: A Review with a Focus on Existing and Potential Options for Control

**DOI:** 10.3390/ani15010097

**Published:** 2025-01-04

**Authors:** Kennedy Mwacalimba, Peter Kimeli, Raymond Tiernan, Erik Mijten, Tetiana Miroshnychenko, Barbara Poulsen Nautrup

**Affiliations:** 1 Outcomes Research, Zoetis, Parsippany, NJ 07054, USA; kennedy.mwacalimba@zoetis.com; 2 Department of Clinical Studies, Faculty of Veterinary Medicine, University of Nairobi, P.O. Box 30197-00100, Nairobi, Kenya; kimeli08@uonbi.ac.ke; 3 Centre of Excellence, Zoetis, D18 T3Y1 Dublin, Ireland; raymond.tiernan@zoetis.com; 4 Zoetis Belgium S.A., 1930 Zaventem, Belgium; erik.mijten@zoetis.com (E.M.); tetiana.miroshnychenko@zoetis.com (T.M.); 5 EAH-Consulting, 52064 Aachen, Germany

**Keywords:** feedlot cattle, sub-Saharan Africa, foot-and-mouth disease, contagious bovine pleuropneumonia, lumpy skin disease, Rift Valley fever, bovine respiratory disease

## Abstract

Livestock production in sub-Saharan Africa (SSA) should address health challenges and boost productivity to reach the projected increase in population growth. This review aimed to define and describe economically important diseases of feedlot cattle with a focus on current therapy and control strategies. Five diseases were selected for review, which represent an ongoing threat to feedlot profitability in SSA. Foot-and-mouth disease is an economically devastating viral disease that severely affects production and trade. Therapy includes local treatment and systemic application of antimicrobial and anti-inflammatory drugs. Vaccines are available, but are limited by uncoordinated vaccine campaigns, poor distribution to remote parts of the countries, and lack of cross-protection among different serotypes and topotypes. Losses due to contagious bovine pleuropneumonia derive from high mortality, morbidity, and trade restrictions. Antimicrobials are used to reduce mortality and limit the spread of disease. Lumpy skin disease leads to losses due to morbidity, mortality, quality degradation of hides, and trade restrictions. Therapy includes topical treatment and the systematic use of antibiotics and anti-inflammatory drugs. Vaccines are available, but vaccine failures and adverse events have been reported. Rift Valley fever is one of the most important arthropod-borne zoonoses in SSA. Outbreaks are treated by mass vaccination, although this may not be coordinated in all SSA countries. Bovine respiratory disease is the most common and costly disease in global cattle production. Losses result from deaths, treatment costs, and reduced growth performance. Efficacious vaccines against the main causative agents are available, but their uptake differs greatly among SSA countries. As eradicating these diseases is not a realistic aim in most SSA countries in the future, prevention and control remain critical. This review identifies areas for potential improvement, including the following. (1) Improvement of biosecurity measures where they are nonexistent or sub-optimal. (2) An increase in vaccine uptake, which could be supported by the introduction of new efficacious vaccines, with fewer side effects and less need for a strict cold chain. (3) Evaluation of newer products regarding their ability to improve health, reduce the spread of disease, and support the prudent use of antimicrobials.

## Simple Summary

This review defines and describes the economically important diseases affecting feedlot cattle in sub-Saharan Africa (SSA) and assesses current therapy and control strategies. Five key diseases—foot-and-mouth disease (FMD), contagious bovine pleuropneumonia (CBPP), lumpy skin disease (LSD), Rift Valley fever (RVF), and bovine respiratory disease (BRD)—present ongoing threats to the profitability of feedlots in SSA. Due to the challenges of eradicating these diseases, prevention and control are crucial to maintaining livestock health and economic stability. This review identifies several areas for improvement: (1) enhancing biosecurity measures where they are inadequate; (2) increasing vaccine uptake, supported by the development of more effective vaccines with fewer side effects and less reliance on strict cold-chain logistics; and (3) evaluating newer therapeutic products for their potential to improve animal health, reduce disease transmission, and encourage the prudent use of antimicrobials. By focusing on these areas, this review underscores the importance of strategic disease management to safeguard the future of cattle production in SSA and enhance feedlot profitability.

## 1. Introduction

A large number of livestock are found in sub-Saharan Africa (SSA), including 20–25% of the world’s ruminants [1]. Livestock production is mostly subsistence and is characterized by low productivity, which may not be enough to meet the existing domestic requirements for animal protein [2]. This is coupled with the highest population growth globally [3]. The current increase in livestock production does not match human population growth, creating a demand deficit and posing great challenges on the continent [4]. Market-oriented livestock production, exemplified by cattle feedlots, has been gradually emerging in Ethiopia and Kenya. The feedlot sector in these countries continues to experience growth and development [2,5]. Feedlots help meet the projected increase in domestic meat demand and offer an opportunity to boost a country’s economy by expanding economically important export markets [2]. The higher productivity in feedlots compared to traditional farming increases the amount and quality of beef and reduces greenhouse gas emissions [6]. Currently, the greenhouse gas emission per unit of livestock is four times greater in eastern Africa than the global average [7]. In South Africa, calculated beef methane emissions for different production systems were 78.9 kg, 62.4 kg, and 58.9 kg CH_4_/head/year in commercial, communal. and feedlot cattle, respectively [8]. Additionally, feedlots successfully reduce livestock vulnerability to climate change and offer a holistic and sustainable approach to addressing climate change challenges in SSA [6].

With an assumed increasing importance of feedlots in SSA, we aimed to identify economically important diseases of feedlot cattle in SSA. This review not only provides an overview of the diseases and their economic impact but also summarizes current treatment and control strategies, thereby identifying gaps in prevention and therapy and potential options for improvement. The review is divided into two parts. The first part explains the identification and selection of economically relevant diseases, which necessitated an understanding of the various types of cattle feedlots currently in SSA. The second part summarizes the main characteristics of these diseases, emphasizing their economic impacts and current treatment and control strategies. Finally, potential options for improving disease treatment and control are suggested based on the review’s findings.

## 2. Characteristics of Sub-Saharan Cattle Feedlots and Selection of Economically Relevant Diseases

### 2.1. Characteristics of Sub-Saharan Africa Cattle Feedlots Using Examples from South Africa, Ethiopia, and Kenya

A commercial feedlot is a confined yard area with watering and feeding facilities where livestock are handled entirely for the purpose of production [2]. The contribution of commercial feedlots to the total cattle supply varies greatly among SSA countries. In Ethiopia and Kenya, the proportions of cattle produced in feedlots have been reported to be <1% [9] and 1% [10], respectively, whereas in South Africa, 65% to 70% of the cattle slaughtered in abattoirs were derived from feedlots [11]. The number of livestock varies across farms depending on their capacity [2]. In Kenya, the capacity varies between fewer than 100 head and several thousand head [5], whereas up to 100,000 head of cattle may be held in one South African feedlot [12].

There are two main systems of cattle ownership in feedlots. In private feedlots, owners purchase or raise the animals themselves. In custom feedlots, the operator rents the facility to external animal owners. In private feedlots, the owner assumes all risks associated with feeding, care, and marketing of the animals. In contrast, in custom feedlots, the operators do not own the animals or manage their care, leaving the risks with the animal owners, who pay for the use of the facilities [13]. In a study on Ethiopian cattle feedlots, 80.6% of the respondents indicated that they fattened the purchased bulls in privately owned barns, whereas 19.4% fattened in rented barns [14]. Cattle derived from feedlots are mainly destined for high-quality meat production, as requested by restaurants or hotels, or are intended for export [2], with Middle East markets being the traditional destination of Ethiopian live cattle and cattle meat [14].

The age and source of animals entering a feedlot also varies greatly in SSA. Animals can be placed in the feedlot at any age, i.e., shortly after weaning, as yearlings, at two and a half years of age, or older [13]. In a study conducted in high-standard feedlots in South Africa, weaner calves were bought between the ages of 5 and 10 months from auctions or farms [15]. In contrast, in Ethiopia, the vast majority of animals (94%) are aged between 4 and 6 years when purchased by commercial feedlots, with pastoralists and smallholders being the suppliers [2]. In Kenya, animals were placed in feedlots from 2 years to over 4 years of age. Younger animals arriving at a feedlot primarily come from three sources: cattle raised by the feedlot owners, steers purchased from ranches, and selected steers from pastoral markets supplied by contracted traders. Older animals, on the other hand, are mainly purchased from pastoral markets. In Kenya, there are no established channels linking producers and feedlot operators, forcing feedlot operators to purchase whatever animals closely meet their requirements, even if they are older (over 4 years) and in poor condition [5].

In addition to traditional feedlots, communally established cattle feedlots have emerged in SSA as a sustainable livelihood option to enhance climate change resilience and food security. Smallholder farmers, who are disproportionately affected by climate change due to limited adaptive capacity and resources, benefit from these communal feedlots. These community-managed facilities offer controlled environments for feeding and animal management [6]. Although communally established cattle feedlots offer a holistic and sustainable approach to addressing climate change challenges, farmers face many constraints in adopting cattle feedlots, such as financial limitations, missing infrastructure, and resource constraints [16].

Just as the characteristics of the feedlots differ, so do the biosecurity measures. Biosecurity includes all measures to prevent the introduction of pathogens and reduce the spread of pathogens in the feedlots [17]. A study investigating the implementation of biosecurity in export-oriented cattle feedlots in Ethiopia revealed that biosecurity measures were deficient at the time. Hygiene practices of the feedlot workers and visitors were nonexistent. Feedlot operators bought all bulls from Borena markets without knowledge about previous health status or the bulls being subjected to any tests or veterinary pre-purchase inspection [14]. The origin of bulls in these markets was mainly Borena pastoral systems [2,18], and the Borena pastoral area has a history of the occurrence of transboundary animal diseases, such as foot-and-mouth disease (FMD), lumpy skin disease (LSD), and contagious bovine pleuropneumonia (CBPP). Nevertheless, most feedlots undertook few or no preventive measures to combat disease transmission, although almost all feedlots (96.8%) had a history of sudden deaths of their bulls and 83.9% of operators indicated that they were concerned about FMD [14]. In the Ethiopian feedlots under investigation, all bulls were vaccinated for FMD, LSD, CBPP, anthrax, black leg, and pasteurellosis as part of the national sanitary and phytosanitary requirements of animal quarantine. However, vaccination began one to three weeks after registration by veterinary authorities, leaving a time gap sufficient for diseases to spread to healthy bulls. Overall, the current biosecurity measures in Ethiopian feedlots have been reported as a potential challenge for the country’s future cattle export opportunities [14].

In South Africa, feedlot characteristics vary substantially. Feedlots characterized by poor or improvable management coexist with feedlots that have introduced high standards and biosecurity measures, comparabe to well-managed feedlots in other parts of the world [15].

### 2.2. Diseases Threatening SSA Feedlot Cattle Profitability

Ethiopian feedlot owners were asked about the main constraints of their daily business. Most feedlot farms noted that the market was considered the most important constraint for commercial fattening. Feed was the second limiting factor, followed by the type or quality of livestock and water shortage. In almost all feedlot farms, diseases were considered minor constraints for commercial business [2]. Similarly, in Kenya, pests and diseases were considered to be low constraints for beef finishing and fattening. The most important constraints in this survey were feed costs, availability of livestock, feed scarcity, and market access [5]. These impressions were underlined by prevalence of diseases such as brucellosis, bovine tuberculosis, or salmonellosis, which were low in feedlots compared to dairy commercials or urban/peri-urban production systems [9].

Nevertheless, export-oriented feedlots have been repeatedly challenged by the risk of transboundary animal diseases (TADs), which can affect or prevent live cattle and meat export [14]. TADs are defined by the Food and Agriculture Organization of the United Nations as highly contagious and transmissible epidemic diseases of livestock that have the capacity for rapid spread to new areas and regions, regardless of national borders, and have severe socio-economic and public health consequences [19]. The prevalence or outbreaks of TADs can result in trade bans, with enormous economic consequences for the affected country [20]. Therefore, this review focuses primarily on TADs, with a particular emphasis on seven diseases that have previously been classified as List A by the World Organization for Animal Health (OIE): bluetongue, contagious bovine pleuropneumonia (CBPP), foot-and-mouth disease (FMD), lumpy skin disease (LSD), Rift Valley fever (RVF), rinderpest, and vesicular stomatitis [21]. Three diseases were excluded from further consideration for the following reasons: bluetongue, as the infection in cattle is typically subclinical [22]; vesicular stomatitis, as the disease is currently limited to the Americas [23]; and rinderpest, which was eradicated in 2011 [24].

In addition to the selected TADs, the bovine respiratory disease (BRD) complex was included in this review, as it has been shown to substantially impact the profitability of feedlots in South Africa, where cattle arrive shortly after weaning [15]. Infectious bovine rhinotracheitis virus, which has been considered the most important pathogen of BRD, can not only erode feedlot profits in all SSA feedlots by depressed animal performance [13] but also represents a constraint on the livestock trade, especially in international markets [25].

Accordingly, the list of diseases for further review included FMD, CBPP, LSD, RVF, and BRD. The main characteristics of the selected diseases are summarized in Table 1 and Table 2. It is important to note that many diseases of great importance for smallholder farmers are missing, such as brucellosis, bovine tuberculosis, salmonellosis, or tick-borne diseases, which can heavily impact the livelihood of these farmers. However, the aim of the review was to summarize diseases of main economic importance in feedlots, where market access has been assessed as the main constraint.

## 3. Main Characteristics of the Selected Diseases

The main characteristics of all diseases are summarized in Table 1 (etiology, epidemiology, and clinical characteristics) and Table 2 (current treatment and prevention strategies).

### 3.1. Foot-and-Mouth Disease

FMD is a highly contagious and economically devastating viral disease of global importance [26]. It deeply affects the production of livestock and disrupts regional and international trade in animals and animal products [27]. FMD is a disease listed by the World Organization for Animal Health (WOAH, formerly OIE) [28].

The disease may have emerged in the Middle Ages and spread from the Mediterranean area to other parts of the world through trade and migration [29]. The current worldwide distribution of FMD virus mirrors economic development, as prosperous countries have eradicated the disease, whereas developing countries lack the resources and infrastructure to do so [30]. The disease is common in Africa, the Middle East, Asia, and some parts of South America [29]. However, the WOAH has certified Botswana, Namibia, and South Africa as FMD-free zones [31]. Countries that are currently free of FMD without vaccination remain under constant threat of new outbreaks [29].

#### 3.1.1. Etiology and Epidemiology

FMD is caused by a virus belonging to the genus Aphtovirus and family Picornaviridae. Seven immunologically distinct serotypes of the FMD virus have been identified, namely, A, O, C, Asia 1, and South African territory (SAT) 1, SAT 2, and SAT 3. Except Asia 1, all serotypes have historically been found in Africa [32], although serotype C is considered extinct [33]. Immunity to one serotype does not cross-protect against the others. A high mutation rate results in numerous and constantly evolving intra-serotypic subtypes or topotypes that may cross-protect incompletely [30,32]. As the FMD virus continues to evolve, it gives rise to new strains, causing periodic upsurges in incidence and increasing the risk of spreading into new areas [34].

FMD affects animals with split hooves, with cattle being the main hosts for the disease and the most vulnerable to infection. Although the virus can infect many wildlife species, such as wild pigs, deer, and antelope, the only confirmed wildlife reservoir is the African buffalo, which acts as a bridge or maintenance host for disease transmission [29]. The virus replicates and persists in African buffalo with minimal disease pathology [30], which is why wildlife plays a unique and important role in the epidemiology of the disease [35].

The virus may occur in all secretions and excretions of acutely infected animals, including expired air. Transmission is generally promoted by direct contact between infected and susceptible animals or more rarely indirect exposure of susceptible animals to the excretions and secretions of infected animals. FMD is considered a negligible zoonotic risk [36].

#### 3.1.2. Clinical Signs

Clinical signs can range from mild or inapparent to severe, depending on the virus strain, exposure dose, animal age, host species, and degree of host immunity. Deaths are uncommon except in young animals, which may die from multifocal myocarditis or starvation. Most adults recover in 2–3 weeks, although secondary infections might delay recovery. Morbidity may approach 100%, whereas mortality is generally low in adult animals (1–5%), but higher in young calves (20% or higher). Clinical signs include pyrexia and vesicles (aphthae) on buccal and nasal mucous membranes and/or between the claws, coronary band, and teats, resulting in smacking of the lips, grinding of the teeth, drooling, lameness, and stamping or kicking of the feet. The vesicles rupture after 24 h, leaving erosions [37].

FMD diagnosis requires laboratory confirmation, usually involving virus isolation and identification via culture and immunoassay. Alternatively, molecular-based techniques can be used to detect the presence of viral nucleic acids [29].

#### 3.1.3. Economic Consequences

FMD places a huge economic burden on the livestock sector in endemic countries. In low- and middle-income countries, FMD exacerbates poverty and constrains development by preventing trade with lucrative markets for animals and animal products in FMD-free countries [38].

The economic impact of FMD results from direct losses due to morbidity and mortality, as well as from indirect losses, including control costs, such as vaccination and diagnostic tests, and denied access to markets. Knight-Jones and Rushton estimated the total annual impact only due to direct losses and vaccination at USD 2 billion in Africa for 2011, i.e., not considering indirect costs due to trade restrictions [39]. However, indirect costs are significant in countries that depend on meat and livestock exports. For instance, in 2011, Ethiopia generated revenues of USD 211.1 million primarily from live animal trade with the Middle East, involving more than 330,000 head of cattle [39]. FMD is one of the major diseases in Ethiopia, hampering the export of livestock and livestock products to the Middle East and other African countries. During the Egyptian trade ban of 2005/2006, Ethiopia lost more than USD 14 million [20]. Uganda’s endemic FMD situation prevents the country from accessing lucrative export markets for livestock and livestock products, and Uganda’s performance in the global export trade is negligible due to FMD [40]. In Zambia, the FMD endemicity has been assumed to be the reason for poor investment in livestock [41].

#### 3.1.4. Treatment and Control Strategies

There is no causal treatment for the disease. However, treating skin lesions with a mild disinfectant and protective dressing in inflamed areas is recommended. Broad-spectrum antibiotics are useful against bacterial infection, and the administration of flunixin meglumine is reported to have an excellent systemic response [42]. The most commonly used antimicrobial in SSA is oxytetracycline. A novel topical anesthetic wound formulation (Tri-Solfen^®^, Medical Ethics Pty Ltd., Melbourne, Australia) that has been developed for treating cattle with FMD is registered in SSA countries. In a field trial, it has been shown to be superior to the administration of parenteral oxytetracycline, thereby representing an alternative non-antimicrobial therapy where available [43].

Preventive vaccination coupled with stamping out cases was adopted by most European countries until 1990, when freedom from FMD was declared and vaccination was terminated in most European countries. In countries that have achieved FMD-free status, strict zoo-sanitary measures are critical, involving import control on animals and their products from affected countries [30]. There have been successes in parts of Africa, for example, Botswana, Namibia, and South Africa have FMD-free zones certified by the WOAH and protected by a vaccination zone surrounding high-risk areas [31]. However, FMD is not usually fatal and often not a priority for control in many developing countries. Therefore, vaccination is used only to a very limited extent, with mass vaccination being beyond the means of many countries, increasing the risk of reservoirs for viral dissemination [30].

Nevertheless, vaccines remain the mainstay for emergency and prophylactic use [30]. Cattle destined for export must be vaccinated against FMD in countries with established sanitary and phytosanitary regulations, such as Ethiopia [14].

Currently, mainly inactivated vaccines containing up to four serotypes are used to prevent FMD in endemic regions. However, the success of vaccination is restricted due to limited cross-protection among different strains and topotypes within the same serotype and the frequent emergence of new variants that may escape vaccine-induced immunity [29]. Additionally, the requirement for a cold chain makes widespread vaccination in developing countries a complex logistical challenge [34]. The vaccines take several days to elicit immunity and need a booster and repeated vaccinations at intervals between 4 and 12 months [30].

Including more virus serotypes in a vaccine in order to enhance effectiveness is costly, thus restricting its use in many developing countries [34]. In Africa, the greatest virus diversity exists, coupled with the least incentive to produce tailored vaccines [30].

However, a new initiative called AgResults has been launched to incentivize the private sector to invest in innovative research and delivery solutions that benefit smallholder farms. As part of this initiative, the AgResults FMD Vaccine Challenge Project aims to support the development and adoption of high-quality quadrivalent foot-and-mouth disease (FMD) vaccines specifically designed to meet the needs of eastern Africa, covering serotypes A, O, SAT1, and SAT2 [34]. A recent publication announced the development of a vaccine candidate that passed the criteria set by the AgResults Vaccine Challenge Project and offers good cross-protection against a panel of regional FMD virus strains [38].

### 3.2. Contagious Bovine Pleuropneumonia

CBPP was previously considered Africa’s second-most important disease after rinderpest [44]. While rinderpest has been successfully eradicated worldwide [24], CBPP continues to plague SSA [45]. It is one of the most important TADs [46], and can cause major productivity losses for the bovine industry due to high mortality and morbidity rates [47] and trade restrictions [48]. CBPP is a listed disease [28] and was the only bacterial disease contained in the former List A diseases of the WOAH (formerly OIE) [21].

CBPP is considered an old disease, as it may have been documented since the second half of the 16th century. Its cradle was in the Central Eastern Alps, and it has spread nearly worldwide due to increased livestock trade. Except for Africa, CBPP has been largely eradicated to date through the implementation of mass vaccination and stamping-out policies [47]. Despite many efforts to effectively control CBPP in Africa, the disease is still endemic in most SSA countries [49], except Namibia (zone located south of the Veterinary Cordon Fence), and South Africa, Botswana, and Eswatini (countrywide) [50]. Between 1995 and 2002, 2612 outbreaks were reported in 12 SSA countries [44].

#### 3.2.1. Etiology and Epidemiology

CBPP is caused by *Mycoplasma mycoides* subsp. mycoides (*Mmm*) [51], previously referred to as the small colony (SC) type of *Mmm* [45].

Under natural conditions, *Mmm* affects cattle (*Bos* genus) and water buffaloes. Although *Mmm* has been isolated in goats and sheep, small ruminants and wild animals do not play a role in the epidemiology of the disease. There is no evidence that humans are infected by CBPP [51].

CBPP is mainly spread by inhalation of droplets from infected coughing animals. Although close and repeated contact is generally considered necessary for transmission, the disease may be transmitted up to 200 m under favorable climatic conditions. Nonclinical bovine carriers with chronic infection may retain viable organisms in encapsulated lung lesions and are an important source of infection. Cattle movements and cattle gatherings are important factors in the spread of the disease [52].

Prevalence tends to be higher in extensive cattle production systems compared to more intensive beef production systems where animals are confined [44]. Prevalence in extensive cattle systems varies greatly in different areas, and levels up to 96% have been reported [53]. In export quarantine feedlots in Ethiopia, prevalence of 8.0% [54], 5.9% [55], and 0.4% [56] has been reported, depending on the origin of the cattle. It was assumed that the low prevalence of 0.4% in one study might have been due to an ongoing annual mass vaccination program and antibiotic treatment at the pastoral production level in the area where the bulls originated [56].

#### 3.2.2. Clinical Signs

The incubation period for naturally infected animals can range from 3 weeks to 6 months, with most cases becoming apparent in 3–8 weeks [52]. The long incubation and the potential subclinical appearance of the disease facilitate the establishment of infection in a herd before it is noticed [47].

CBPP is a respiratory disease characterized by pneumonia and serofibrinous pleurisy [57]. There are four main clinical forms that have been described [47,57]. The hyperacute form is rare and occurs during the onset of an outbreak. Death may occur without previous clinical signs of pneumonia. The acute form commonly occurs at the beginning of an epidemic and is characterized by high fever and severe respiratory distress that can be further exacerbated by exercise. As the disease progresses, cattle can show tachypnea, chest pain with grunting, coughing, subcutaneous edema, catarrhal to purulent nasal discharge, and ptyalism. The mortality rate in the acute phase can reach 50%, but varies according to the severity of the disease. While complete recovery is possible, many cases will develop into a chronic form, which is more common towards the end of an epidemic. The affected animals might show unspectacular signs with mild respiratory distress on exercise and low-grade fever, but they can also exhibit a violent and prolonged cough. The animals may remain in poor condition for an extended period, depending on the size of the chronic lung lesions. Subacute and symptomless forms of CBPP occur frequently and are characterized by mild or no clinical signs at all. However, these animals are able to transmit the disease, thereby playing an important role in the maintenance and epidemiology of the disease [47,57].

Morbidity rates vary significantly between herds [44] and can reach 100% in susceptible herds that have never been challenged by CBPP [49]. Total mortality can reach 75–90% in epidemic outbreaks, but is usually <10% in enzootic regions [45].

Although severe respiratory symptoms can also be observed in young cattle, *Mmm* shows a distinct tropism towards the joints in cattle up to 6 months, with carpal and tarsal joints being most frequently affected by synovitis [47,57].

Diagnosis of CBPP relies on clinical examination, postmortem inspections, and laboratory analyses [49]. CBPP is challenging to diagnose based on clinical signs alone, as there can be many causes of severe pneumonia in cattle. It should be taken into account in a herd that exhibits pneumonia in adults and polyarthritis in calves [46]. Serological analyses are commonly used for disease investigation in Africa on a herd level, but are not useful for diagnosis in individual cattle, where polymerase chain reaction (PCR) tests have proven useful [47,49].

#### 3.2.3. Economic Consequences

In affected countries, economic losses are experienced due to the death of animals and low performance during convalescence [58]. Additional costs accrue from vaccination and treatment. Indirect losses result from lost market opportunities through trade restrictions, quarantine costs, and delayed marketing [44]. Ethiopia has lost substantial market share and foreign exchange earnings over the last decades due to frequent bans by Middle East countries [53]. A modeling study estimated the total economic losses that could be avoided if CBPP were controlled through vaccination and antibiotic treatment in 12 sub-Saharan countries. The avoidable losses were calculated to be EUR 45 million, with an average loss per country of EUR 3.7 million, ranging from EUR 0.765 million in Ghana to approximately EUR 15 million in Ethiopia. The study considered losses in cattle and cattle products due to mortality and morbidity (production losses), accounting for two-thirds of total losses. The estimates can be regarded as a significant underestimation of total losses, as indirect losses resulting from reduced fertility, lost market opportunities through trade bans, quarantine costs, and delayed marketing were not considered due to data limitations [44].

#### 3.2.4. Treatment and Control Strategies

Antibiotics are recommended only in endemic areas, because the organisms may not be eliminated and carriers might develop [58]. Nevertheless, antibiotic treatment for clinical CBPP is standard field practice in many African countries. Antibiotics that are effective against *mycoplasma* reduce the clinical signs and shedding of bacteria, but do not allow a complete cure. The WOAH advocates the prudent use of antibiotics when slaughter cannot be achieved [52]. A feasible treatment regime can reduce transmission by decreasing infection duration and *Mmm*’s effective reproductive numbers [44]. Mariner et al. (2006) estimated in a model of CBPP transmission dynamics in eastern Africa that a 50% reduction in the length of the infected period would cause a 60% decline in the number of cases and a 73% decline in mortality. The authors concluded that research on the efficacy of prudent and feasible treatment regimens should warrant the same attention as vaccine development [59].

In Africa, cattle owners often treat their sick animals with tylosin and oxytetracyclines when observing respiratory symptoms, including signs of CBPP [60]. Further commonly used antibiotics include tetracyclines, erythromycin, lincomycin, spectinomycin, and tilmicosin, although resistance to some of these antimicrobials has been noted [46]. Studies have also demonstrated the therapeutic efficacy of macrolides and fluoroquinolones [47]. Danofloxacin has been shown to reduce the spread of CBPP to healthy in-contact cattle [61]. Two studies have been carried out in Kenya and Zambia, assessing the effectiveness of two new-generation macrolides (tulathromycin and gamithromycin) compared to oxytetracyclines and no antibiotic use against different challenge strains of CBPP [62]. Using the mean adapted Hudson and Turner score, which includes clinical signs, postmortem findings, and serology, tulathromycin protected 82% and gamithromycin 56% and oxytetracycline 80% of the animals in Kenya, whereas in Zambia, protection rates were 98% (tulathromycin), 94% (gamithromycin), and 80% (oxytetracycline) [62].

Four essential control approaches have been defined for the mitigation of CBPP in Africa: vaccination, treatment, movement control, and stamping out through slaughter with compensation. Unfortunately, most of the CBPP-affected countries in Africa do not apply all of these measures simultaneously due to economic and logistic reasons, but restrict the control of CBPP to vaccination and treatment [44].

Cattle destined for export must be vaccinated against CBPP in countries with established sanitary and phytosanitary regulations, such as Ethiopia [14].

The only vaccines commonly used today are T1/44 and T1sr, which are produced with attenuated *Mmm* strains. Both vaccines are characterized by poor and variable efficacy, with only 30% to 60% of vaccinated animals being protected. T1/44 induces a 1-year long protection, and the vaccine can induce good immunity when herds are revaccinated annually, with the level of protection exceeding 85%. However, T1/44 may have residual virulence upon primary vaccination, and systemic or local adverse reactions are common. T1sr has no virulence left, but can afford only 6 months of protection [52,53].

Unfortunately, vaccine quality has declined for several reasons. Some African manufacturers lack independent quality control, and vaccines are often mishandled during inoculation due to poor cold chain maintenance. Additionally, vaccines may contain sub-optimal quantities of *Mmm* strains. These issues have led to post-vaccination reactions and occasional deaths, causing many pastoralists to refuse vaccination altogether [49].

### 3.3. Lumpy Skin Disease

LSD is an economically significant TAD [63]. Because of its economic losses and the potential for rapid spread, LSD is a disease listed by the WOAH [28].

Lumpy skin disease was first diagnosed in Zambia in 1929, and it spread quickly in the cattle community throughout Africa. Today, it is endemic in most African countries, some countries in the Middle East, Eastern Europe, Russia, and China [45]. However, there is a growing risk of it spreading into other geographic regions due to evidence suggesting that insect vectors contribute to transmission, the effects of global climate change [64], and shifts in animal and animal product trading patterns [65].

#### 3.3.1. Etiology and Epidemiology

LSD is caused by the LSD virus, belonging to the genus *Capripoxvirus* of the *Poxviridae* family. It is genetically related to sheep pox and goat pox viruses [66]. The LSD virus affects cattle and water buffalo [67]. Although it can affect wild ruminants, the role of wildlife in the epidemiology of LSD is not yet well understood [66].

The LSD virus is transmitted mainly by arthropod vectors [63], although a potential role of hard tick vectors has also been reported [68]. The transmission is apparently purely mechanical rather than biological, and the virus generally does not survive in vectors for long periods [63]. The vectors mainly become infected from skin lesions of affected animals, where the virus can persist in necrotic skin nodules for up to 33 days or longer, in desiccated crusts for up to 35 days, and at least 18 days in air-dried hides [66]. As nodules are also found on mucous membranes of the eyes, nose, mouth, rectum, udder, and genitalia, the virus is also present in blood, nasal and lachrymal secretions, milk, semen, and saliva when the nodules ulcerate [69]. Indirect infection through infective saliva and contaminated feed and water has been reported, whereas direct transmission seems to be of low importance, at least in the early stages of disease, as no positive correlation between cattle density and infection rates were shown [66].

Outbreaks of LSD are highly associated with a seasonal peak of mechanical vectors in wet and warm weather conditions. However, the movement of infected stock plays an important role in rapidly spreading LSD over large areas [63].

#### 3.3.2. Clinical Signs

In general, outbreaks are more severe, with higher morbidity rates during the initial introduction of the infection to a region not previously affected by LSD compared to infections in endemic areas [69]. The morbidity rate varies between 5% and 45%, but can be up to 100%, whereas the mortality rate is usually under 10%, but may reach 40% [66]. Investigations of two outbreaks in Ethiopia between September 2011 and April 2012 revealed higher morbidity and mortality rates in feedlot cattle than in extensive cattle production (morbidity 14.1% versus 8.2%, mortality 5.26% versus 1.65% for feedlot and extensive production system, respectively), although most cattle in feedlots were vaccinated against LSD [70].

The initial clinical signs after an incubation phase of 2 to 5 weeks include fever, lacrimation, increased nasal and pharyngeal secretions, depression, and anorexia. These are followed by nodular skin lesions of varying extent affecting the entire animal, with the preferred locations being the head, neck, limbs, genitalia, and udder [71]. The raised nodular lesions are usually approximately 1–7 cm in size, with smaller nodules often coalescing to form larger lesions [72]. After 2–3 weeks, nodules ulcerate and become necrotic [73]. The necrotic cores, which are often known as “sit-fast,” detach from the surrounding skin [71]. In severe cases, ulcerative lesions appear in the mucous membranes, such as the eye and nasal cavities, resulting in excessive salivation, lachrymation, and nasal discharge. After at least 1 month, the ulcerations heal and the skin becomes thick and hyperpigmented [73]. However, skin lesions cause permanent damage to the hides [45]. In severe cases, complications such as keratitis, dysentery, lameness, pneumonia, and myiasis can occur [66]. Animals that recover may remain in extremely poor body condition for up to 6 months [63]. After recovery from natural infection, the cattle do not become carriers [64] and will have lifelong protection [74].

Diagnosis of LSD is based on clinical signs with laboratory confirmation by virus isolation, polymerase chain reaction, and electron microscopy. However, the field diagnosis is often based on the characteristic clinical signs of the disease [69].

#### 3.3.3. Economic Consequences

LSD causes severe economic losses, mainly due to the prolonged effect on productivity associated with chronic debility in infected cattle. Additionally, economic losses arise from the degradation in quality of skin and hides, culling and mortalities, cost of treatment and prevention of the disease, movement controls, and exclusion from access to specific export markets [69].

During two outbreaks in Ethiopia between September 2011 and April 2012, direct losses from mortalities were estimated at USD 51,590. When considering only the mortalities in the nine feedlots with a total of 1992 cattle included in the investigation, losses were calculated as USD 50,159, not including higher additional feed costs to assist sick animals during their lengthened recovery and lower prices received for cattle not suitable for export [70].

A study on 20 export-oriented feedlots in Ethiopia estimated the direct economic losses caused by LSD. The feedlots purchased the bulls predominantly from Borena markets, with bulls originating from pastoral systems. All bulls were vaccinated, starting a week after the final bull entered the feedlots. However, the duration from the introduction of the first batch of bulls to vaccination was sufficient to allow the spread of disease to susceptible bulls. During the approximately half-year observation period, 6.1% of bulls showed clinical signs and lesions suggestive of LSD and 1.8% of bulls were found dead from LSD. The economic losses, considering mortalities and rejection rate from international markets, were calculated at USD 667,785.60 (as of 2011). However, the authors noted that the real losses can be expected to be higher, since feedlot operators incurred additional costs associated with treating sick bulls and maintaining rejected animals [18].

#### 3.3.4. Treatment and Control Strategies

Because LSD is a viral illness, there is currently no specific treatment. Treatment is only symptomatic. Antibiotics such as penicillins, cephalosporins, tetracyclines, and fluoroquinolones are used to prevent or treat subsequent bacterial infection, and anti-inflammatory drugs are used to reduce inflammation and fever and provide comfort. Additionally, the local application of ointment or antiseptic sprays is useful for wound healing. Intravenous fluid therapy and supportive medications are given in cases of dehydration or anorexia. However, treating LSD is costly and does not always result in full recovery [71,72].

Vector control with regular application of pour-on insect repellents and insecticides can be viewed as a supportive rather than a preventive measure, because it cannot stop LSD from spreading [71].

To prevent the introduction of LSD into a new area, an infected animal must be identified as early as possible. Quarantine of the area and slaughtering of diseased animals and any in-contact animals are necessary, with proper disposal of the animals and disinfection of equipment. Movement controls of animals and animal products as well as mass vaccination are the major control and preventive strategies [74]. However, in endemic countries, vaccination is often considered the only economically feasible way to control the spread of LSD [69]. Cattle destined for export must be vaccinated against LSD in countries with established sanitary and phytosanitary regulations, such as Ethiopia [14].

Because of antigenic homology and cross-protection between sheep pox, goat pox, and LSD viruses, any of these viruses can be used as a vaccine strain [74]. From four available, cheap, live attenuated vaccines [69], two have been widely used in SSA. The South African Neethling LSD virus strain has been used mostly in Africa, followed by the heterologous Kenyan sheep and goat pox strain (KS-1). Protective immunity will develop 10 to 21 days post-vaccination, requiring an annual booster [74]. The Neethling vaccines are reported to have an effective control success of 80% in livestock [73]. Although widely used in the face of outbreaks in Africa, vaccine breakdown and infection of vaccinated animals have been reported [75]. In export-oriented Ethiopian feedlots, an overall incidence of 6.1% was found in vaccinated bulls [76]. However, investigations of reported Neethling vaccine breakdowns or failures have identified various causes, including vaccination of animals that are already in the incubation phase, diagnosis failure, and infrequent or improper use of the vaccine [77]. The vaccine can cause adverse events, often referred to as “Neethling responses” [72], and the skin reactions can affect the cattle owner’s attitude and should be well explained to prevent their reticence to have their animals vaccinated [78]. Heterologous (sheep and goat pox virus-based) vaccines are generally considered to induce fewer side effects, but are less effective [79].

### 3.4. Rift Valley Fever

RVF is one of Africa’s most important human and veterinary arthropod-borne diseases, posing a significant global threat to livestock marketing and human health [80]. It causes major epidemics at irregular intervals of 5–20 years [81], usually when ecological and climatic factors favor competent vector emergence in epidemiological settings [80].

It was first identified during an outbreak of abortion and death in sheep and illness in humans that occurred in the Rift Valley of Kenya after heavy rainfall in 1930–1931. The geographic distribution of the virus has since grown significantly, and now RVF is endemic in most countries on the African continent and the Arabian Peninsula [81,82]. Due to the high numbers of competent vector species present in currently RVF-free regions, the intensification of international trade in live animals, and the impact of climate change, there is a risk of introducing the virus into RVF-free countries [83]. Rift Valley fever is a disease listed by the WOAH [28].

#### 3.4.1. Etiology and Epidemiology

Rift Valley fever is caused by a virus of the order *Bunyavirales*, family *Phenuiviridae*, genus *Phlebovirus* [82]. The virus affects both humans and ruminants, with sheep being the species most susceptible to RVF infection [84]. Although antibodies have been detected in wildlife, no evidence suggests that these animals develop sufficiently high viremia to transmit to mosquitoes [85].

In animals, the RVF virus is transmitted mainly by mosquitos. More than 65 species are described as potential vectors, the vast majority from *Aedes* and *Culex* spp. [85]. The virus is maintained through horizontal transmission between vertebrate hosts and blood-feeding mosquitoes and vertically through infected mosquitoes and their offspring [80,86]. The potential of vertical transmission supports the suggestion that the virus survives for a long period in *Aedes* eggs laid at the edge of a usually dry depression (endemic cycle). When it floods during heavy rains, the eggs hatch and the mosquitos emerge, initiating the epidemic cycle. The dormant survival of the virus in eggs of the mosquitos could also help to explain the cyclic occurrence of epidemics at 5- to 20-year intervals in drier areas [81,85,87]. The epizootic cycles are further driven by the subsequent elevation of various *Culex* mosquito populations, which serve as excellent secondary vectors [87]. Accordingly, *Aedes* spp. mosquitoes were classified as reservoir or maintenance vectors, whereas *Culex* spp. were considered epidemic or amplifying vectors. Transmission in animals can also occur via direct contact with infected animal tissue, bodily fluids, and fomites, particularly if associated with abortions. The relative importance of each mode of transmission varies according to the stage of the epizootic, as in the first stage, the bites of infected mosquitoes are the predominant mode of transmission, whereas direct contact may become more important during the amplification stage of the epizootic [83]. No direct human-to-human transmission has been documented, and mosquito bites do not seem to play a major role [85], as transmission occurs mainly by direct contact with infected tissues or bodily fluids [83]. Involvement in birthing, skinning, or slaughter of infected animals, handling of sick animals, or contact with aborted animal tissue, as well as consumption of undercooked or raw animal products were identified as risk factors for infection of humans [88].

The prevalence of antibodies against the RVF virus in feedlots in central South Africa was 26.2% during an interepidemic period, i.e., four years after the last outbreak, indicating likely viral circulation during the post-epidemic period. No apparent clinical signs of RVF were observed on the farms during the study [89]. However, low-level circulation of the RVF virus in livestock can occur without causing disease outbreaks [90].

#### 3.4.2. Clinical Signs

Rift Valley fever epizootics mainly occur during periods of exceptionally heavy rains, where subsequent flooding results in large increases in mosquito populations [85]. The classical hallmark of RVF epizootics is the large number of near-simultaneous abortions among pregnant ruminants of different stages of pregnancy, which are also known as “abortion storms,” accompanied by deaths of young animals and human cases [82,83,91].

In non-pregnant animals, the virus-induced lesions occur predominantly in the liver [92]. After an incubation period of 1 to 3 days, calves experience fever (40–41 °C), inappetence, weakness, depression, diarrhea, and jaundice, whereas adult cattle often have inapparent infections. However, fever, dull coat, lacrimation, nasal discharge, salivation, anorexia, and bloody diarrhea can occur [93]. The mortality rate in cattle is usually less than 10% [82], but it can be up to 100% in newborn ruminants [80].

In humans, most infections with RVF cause influenza-like symptoms [94]. However, in RVF epidemics, an estimated 1–2% of infections result in severe disease, often with high levels of mortality [85], which are associated with a wide range of clinical signs, including hepatitis, retinitis, delayed-onset encephalitis, and hemorrhagic disease [83].

The sudden onset of large numbers of abortions (“abortion storms”) and mortalities among young animals in affected livestock, together with the appearance of the disease in humans, is considered characteristic of an RVF epidemic [95]. However, clinical diagnosis alone cannot be considered reliable, as the infection of susceptible adult non-pregnant ruminants is often subclinical and hence outbreaks outside the calving season can be easily missed [84]. Current surveillance is sub-optimal in numerous at-risk countries, and therefore many outbreaks remain unreported [85]. Experiences from recent outbreaks in Africa suggest that an outbreak of RVF in livestock is only recognized after diagnosis of the disease in humans [83].

#### 3.4.3. Economic Consequences

Imports of animals and fresh meat from countries or zones not free of RVF are complicated by the recommendations of the WOAH Terrestrial Health Code, which foresees obtaining an international veterinary certificate justifying the absence of infection in the animals or meat [88]. The animals must be kept in quarantine for 30 days before shipment and must not show clinical signs of RVF. Animals must also be vaccinated, complying with the standards described by the WOAH manual, or additional diagnostic tests for RVF are mandatory to exclude an infection with RVF [96].

During outbreaks, economic losses of RVF are significant, resulting from livestock losses, trading bans, and prevention, control, and surveillance measures [94].

Between 1999 and 2023, 39 countries in Africa and the Arabian Peninsula reported outbreaks involving approximately 1000 confirmed human deaths [97]. During those outbreaks, embargoes were placed on livestock exportation, which were shown to have the greatest economic impact on the livestock industry and national economics. In Somalia, for example, the local and/or international bans on livestock trade during the 2006–2007 RVF outbreak were responsible for 64% of the economic burden incurred from the disease. In a scoping review, the estimated total losses due to outbreaks of RVF in different countries ranged from USD 5 to USD 470 million per country [94].

#### 3.4.4. Treatment and Control Strategies

There is no specific treatment for Rift Valley fever. Recovered animals maintain lifelong immunity [85,98].

Control measures usually include vaccination, the control of livestock movements, and vector control, with an emphasis on larvicides in vector breeding sites rather than aerials targeting adults [96]. However, controlling vectors is deemed an unrealistic aim because of the great diversity of competent vectors with distinct ecological behaviors [88].

Mass vaccination is commonly performed when new outbreaks occur. However, the cost of sustained vaccination campaigns against RVF is beyond the capacity of most countries suffering regular outbreaks. Except for South Africa, affected countries in SSA do not practice routine vaccination. Outbreaks of RVF usually occur at irregular intervals, resulting in long interepizootic periods when mass vaccination is ceased [98].

Presently, there is no licensed RVF vaccine for human use, although vaccines are under development [90]. Currently available RVF vaccines for animals are either live attenuated or inactivated vaccines [82]. The first and one of the most widely used livestock RVF vaccine is the live attenuated Smithburn vaccine, developed in 1949. Despite its relatively low cost and ability to induce long-lasting immunity after a single administration, numerous safety concerns associated with its use have been raised, as it can cause harmful changes in internal organs and high abortion rates in pregnant animals [90], and therefore is not recommended for use in pregnant animals [91]. The potential for reversion to virulence has meant that the vaccine is contraindicated in areas where RVF virus has not been detected [84].

A second live attenuated vaccine, Clone 13, has also been registered since 2010 in several SSA countries [91]. The vaccine has been extensively used in South Africa. Published efficacy and safety studies of the Clone 13 vaccine have shown that the vaccine protects animals properly without inducing undesirable clinical signs. However, some safety studies raised concern about the possibility of genetic reassortment between the vaccine and virulent strains, and more recent studies also reported that the vaccine virus crossed the placental barrier, resulting in malformations and stillbirth in sheep [98].

Inactivated vaccines are also available in African countries, although the availability differs between countries. These vaccines require two primary doses and annual booster doses. They can also be used in pregnant animals. Several promising vaccine candidates are currently being developed, including subunit vaccines, virus vectors, and replicon vaccines [91].

### 3.5. Bovine Respiratory Disease

BRD is one of the largest challenges facing the modern-day beef cattle industry [99]. Economic losses result from animal deaths, treatment costs, and reduced growth performance. Although many viruses and bacteria are involved in the outbreak of BRD, the infectious bovine rhinotracheitis (IBR) virus or bovine herpesvirus type 1 is considered to be the most important of the respiratory viruses in feedlot cattle [15]. Despite rigorous IBR control measures, the disease is prevalent in many developed and developing countries [25].

#### 3.5.1. Etiology and Epidemiology

Many viruses and bacteria have been associated with or isolated in BRD cases, and single infections of these viruses are rarely observed in the feedlot. IBR virus, parainfluenza 3 (PI-3), bovine viral diarrhea virus (BVDV), and bovine respiratory syncytial virus (BRSV) are the common viruses associated with acute BRD. Although these viruses can cause respiratory disease without significant interaction with other pathogens, they have been shown to potentiate each other, causing more severe clinical signs and lesions than infection with any of these viruses alone [15]. Positive associations exist between the viruses: being seropositive for any of these viruses means that an individual is more likely to be seropositive for other viruses [100].

The viruses impair the host’s immune defense, predisposing the animal to secondary bacterial infections. The most common bacteria include *Mannheimia haemolytica* (formerly *Pasteurella haemolytica*), *Pasteurella multocida*, *Histophilus somni*, *Arcanobacterium pyogenes*, *Fusobacterium necropherum*, and *Mycoplasma* spp. [101].

BRD is usually observed in cattle within four weeks of transportation to a feedlot [99]. On arrival, animals from various sources are co-mingled, exposing susceptible cattle to infectious respiratory pathogens. Additionally, the immune systems of the cattle are greatly compromised due to stressors, such as weaning, transportation, dehydration, starvation, fatigue, processing, temperature fluctuations, and co-mingling [15].

Additional predisposing factors include dietary changes from pasture to feed of higher energy, as well as extreme temperatures and abrupt changes in temperature and humidity. A dusty environment also increases the risk of BRD, as dust particles reaching the alveoli can exhaust the phagocytic capabilities of the alveolar macrophages and reduce mucociliary clearance [15].

Transmission occurs by direct contact, aerosol, and exposure to contaminated fomites in the animal’s environment. Shared water troughs were reported to be a possible mechanism as virus from infected animals may be transmitted to animals in adjoining pens [102].

The most important feature of IBR is that cattle become latent carriers of the virus after clinical infections. The virus is transported to the trigeminal and sacral ganglia, where it establishes a lifelong latent infection. Stressful conditions can reactivate the virus. Latently infected cattle are considered carriers of the virus, and represent a common infection source [25].

#### 3.5.2. Clinical Signs

A typical clinical case is characterized by high fever, depression, bilateral mucopurulent nasal discharge, gaunt abdomen with rumen atony, coughing, varying degrees of polypnea and dyspnea, and evidence of bronchopneumonia on auscultation. After a few days, dyspnea may become worse, commonly with an expiratory grunt. Mild diarrhea may be present in some cases. If treated early, affected cattle recover within 24–48 h, but severe cases may die despite treatment. Some cattle may recover spontaneously without treatment. Not all cattle with respiratory disease have overt clinical signs of disease. Serologic examinations of feedlot cattle have shown that subclinical infections with bacterial and viral respiratory pathogens are common [15].

#### 3.5.3. Economic Consequences

Bovine respiratory disease is one of the most common and costly diseases in the modern-day beef cattle industry, especially in newly derived feedlot calves [99]. Not only does the disease result in increased medication costs and death, but morbid beef cattle also grow more slowly, develop less efficient feed conversation ratios, and tend to need additional time to reach carcass quality similar to clinically healthy calves [103].

A study in two South African feedlots reported that lung lesions were present at slaughter in 42.8% of all cattle, of which 69.5% had never been treated for BRD, indicating that many cases were either subclinical or were missed. Using a combined case definition, i.e., animals treated for BRD and/or showing lung lesions at slaughter, the estimated overall incidence was 52.5%. Clinical and subclinical BRD reduced the average daily gain (ADG) of the feedlot cattle, resulting in an overall reduction of 24 g in ADG and an increase of 5.1 days on feed. The hidden costs of reduced growth rate due to BRD was calculated at USD 3.41 per calf with clinical or subclinical BRD. Extrapolating these results to the entire country, the authors calculated annual losses of approximately USD 2.42 million to the South African feedlot industry, not including direct medicine and labor costs or losses due to BRD-associated mortality [104].

The two feedlots included in the study had high management standards, and no mortalities occurred due to BRD during the study period. However, animals were selected to be treated at the earliest sign of respiratory disease, resulting in low mortality. In feedlots with poorer management, pulling has been reported to occur late or inaccurately, and mortalities due to BRD complex can be high, resulting in much higher losses [15].

#### 3.5.4. Treatment and Control Strategies

There is no specific antiviral treatment. Antimicrobials are commonly used to treat and prevent the spread of bacteria. Long-acting formulations of tetracyclines are often used as blanket prophylaxis on arrival/processing in feedlots in South Africa [101]. High-standard feedlots included in a clinical study in South Africa had implemented BRD treatment protocols, which were pharmacologically based on oxytetracycline and tylosin followed by trimethoprim–sulfadiazine [104].

Anti-inflammatory and non-steroidal drugs are used to relieve pyrexia and respiratory symptoms [25].

Prevention and control are mainly based on biosecurity measures. A quarantine of newly purchased animals for 2–3 weeks should be implemented [25], although this is not common practice in many SSA feedlots [14]. Any measure intended to reduce stress for the animal can be supportive. Preconditioning of calves, including vaccination, treatment of parasites, and keeping the calves in extensive grazing camps for 45 days before entering a South African feedlot, resulted in significantly lower morbidity [105]. Vaccines are available against the main viruses involved in BRD. In high-standard feedlots in South Africa, cattle are routinely vaccinated against IBR, BVD, PI-3, and BRSV on day 1 after arrival at the feedlots [15]. However, no vaccination against any of these viral pathogens was effected in Ethiopian export quarantine feedlots [14]. Even vaccination against the most important cause of BRD, the IBR virus, is not common practice in Ethiopia [106].

## 4. Conclusions

A complete eradication of the economically important diseases in cattle feedlots considered in this review does not represent a realistic aim for all SSA countries in the future. Therefore, the focus still has to remain on prevention and control. Taking into account the situation as outlined in this review, the following key areas for support have been identified. (1) The biosecurity should be improved in those feedlots where biosecurity measures are nonexistent or sub-optimal, also including an intensification and/or optimization of vaccination where appropriate. (2) An increase in vaccine uptake could be supported by developing new vaccines with better efficacy, fewer side effects, and less dependency on a strict cold chain. (3) Newer antibiotics should be evaluated on their ability to improve health and reduce the spread of the disease (Table 3).

Additionally, further research is needed to fill current gaps in knowledge on the prevalence and economic impact of the diseases, in order to identify those diseases of most importance for the development of new control and treatment strategies.

## Figures and Tables

**Table 1:** Summary of the etiology, epidemiology, and clinical characteristics of selected diseases of economic importance in feedlot cattle in sub-Saharan Africa.

Disease	Causative Agent(s)	Distribution	Main Clinical Features	Morbidity and Mortality
Foot-and-mouth disease (FMD)	FMD virus (serotypes A, O, South African territory (SAT) 1, SAT 2, SAT 3)	- Endemic in many sub-Saharan African countries - South Africa, Namibia, Botswana certified FMD-free zones	- Pyrexia - Aphthae on buccal and nasal mucous membranes, teats, between claws and coronary band	- Morbidity may approach 100% - Mortality 1–5% in adult animals, 20% or higher in young calves
Contagious bovine pleuropneumonia (CBPP)	*Mycoplasma mycoides* subsp. *mycoides*	- Endemic in many sub-Saharan African countries - South Africa, Botswana, Eswatini (countrywide CBPP-free), Namibia (CBPP-free south of the Veterinary Cordon Fence)	- Respiratory disease, characterized by pneumonia and serofibrinous pleurisy - In calves, synovitis in carpal and tarsal joints	- Morbidity rates vary significantly between herds and can reach 100% in susceptible herds that have never been challenged by CBPP - Mortality can reach 75–90% in epidemic outbreaks, but is usually <10% in enzootic regions
Lumpy skin disease (LSD)	LSD virus (genus Capripoxvirus, family Poxviridae)	- Enzootic in most sub-Saharan Africa countries	- Fever, lacrimation, nasal discharge - Nodular skin lesions, which cause permanent damage to the hides	- Morbidity between 5% and 45%, but may approach 100% in epizootics - Mortality <10%, but may reach 40% in epizootics
Rift Valley fever (RVF)	RVF virus (genus Phlebovirus, family Phenuiviridae)	- Endemic in many sub-Saharan African countries, with epidemics at irregular intervals of 5–20 years	- The classical hallmark of RVF are so-called abortion storms, accompanied by death of young animals and human cases	- Mortality is usually <10%, but may reach 100% in newborn calves
Bovine respiratory disease complex (BRD)	Herpes virus 1 (infectious bovine rhinotracheitis virus), Parainfluenza 3 virus, bovine viral diarrhea virus, bovine respiratory syncytial virus *Mannheimia haemolytica*, *Pasteurella multocida*, *Haemophilus somni*, *Mycoplasma* spp.	- Endemic in most sub-Saharan African countries	- Clinical cases are characterized by fever, coughing, inappetence, depression, tachypnea, and serous nasal and ocular discharges	- In 2 South African feedlots, incidence of BRD was calculated as 52.5%

**Table 2:** Summary of current treatment and prevention strategies of selected diseases of economic importance in feedlot cattle in sub-Saharan Africa.

Disease	Current Treatment	Vaccination	Current Application of Preventive Measures
Foot-and-mouth disease	- Local treatment of skin lesions - Systemic treatment with antibiotics and flunixin meglumine - The most commonly used antimicrobial is oxytetracycline	- Inactivated vaccines with a maximum of 3 serotypes are available	- In many African countries, vaccination is used only to a very limited extent, as mass vaccination is beyond the means of many countries - Eradication achieved in South Africa, Botswana, and Namibia, certified as FMD-free zones - In Ethiopia, vaccination is part of the sanitary and phytosanitary measures, i.e., all cattle destined for export must be vaccinated
Contagious bovine pleuropneumonia	- Antibiotic treatment (mainly tylosin and oxytetracycline)	- Two vaccines produced with attenuated *Mmm* strains are commonly used: T1/44 and T1sr	- Vaccination and treatment are currently the main measures applied in sub-Saharan Africa - In Ethiopia, vaccination is part of sanitary and phytosanitary measures, i.e., all cattle destined for export must be vaccinated
Lumpy skin disease	- Antibiotic treatment to prevent bacterial superinfection, including penicillins, cephalosporins, tetracyclines, and fluoroquinolones - Anti-inflammatory drugs and supportive treatment - Local treatment of skin lesions	- The live attenuated homologue Neethling vaccine has been widely used, followed by the heterologous Kenyan sheep and goat pox strain (KS-1)	- In endemic countries, vaccination is considered the only economically feasible measure - In Ethiopia, vaccination is part of sanitary and phytosanitary measures, i.e., all cattle destined for export must be vaccinated
Rift Valley fever	- There is no specific treatment	- The live attenuated Smithburn and Clone 13 vaccines have been widely used, and inactivated vaccines are available in some African countries	- Mass vaccination is commonly performed with new outbreaks, but in most sub-Saharan countries, it ceases after the outbreak is controlled
Bovine respiratory disease complex (including infectious bovine rhinotracheitis (IBR))	- Antibiotic treatment (e.g., long-acting tetracyclines, oxytetracyclines, tylosin, trimethoprim–sulfadiazine) - Anti-inflammatory drugs for relief of pyrexia and respiratory symptoms	- Vaccines are available against herpes virus 1 (IBR virus), parainfluenza 3 virus, bovine viral diarrhea virus, and bovine respiratory syncytial virus	- In high-standard feedlots in South Africa, cattle were routinely vaccinated against IBR, BVD, PI-3, and BRSV - No vaccination against any of these viral pathogens was required as part of sanitary and phytosanitary requirements of animal quarantine in Ethiopia, and vaccination against IBR is not common practice

**Table 3:** Summary of current limitations or gaps in biosecurity measures, vaccines, and treatment identified for the selected diseases in in sub-Saharan Africa.

Disease	Potential Gaps with Biosecurity Measures	Limitations of Existing Vaccines	Potential Gaps with Current Treatment Options
Foot-and-mouth disease (FMD)	- In Ethiopia, many feedlots buy bulls from Borena pastoral systems (on Borena markets), which have a history of FMD, without any knowledge of health status or veterinary inspection - Although vaccination against FMD is part of national sanitary and phytosanitary requirements of animal quarantine for export markets, vaccination often starts too late to prevent transmission	- Mainly inactivated vaccines are used, containing up to 3 serotypes (from a total of 5 serotypes found in Africa) - Limited efficacy, as cross-protection among different strains and topotypes varies greatly and new variants frequently emerge	- No causal treatment; “old” antimicrobials, such as oxytetracycline are most commonly used
Contagious bovine pleuropneumonia (CBPP)	- In Ethiopia, many feedlots buy bulls from Borena pastoral systems (on Borena markets), which have a history of CBPP, without any knowledge of health status or veterinary inspection - Vaccination against CBPP is part of national sanitary and phytosanitary requirements of animal quarantine for export markets; however, vaccination often starts too late to prevent transmission	- The two vaccines (T1/44 and T1sr) commonly used today are characterized by poor and variable efficacy, and systemic or local adverse effects are common with T1/44	- Mainly “old” antimicrobials are regularly used, such as tylosin and oxytetracycline - Although a study reported higher protection rates with new-generation macrolides (tulathromycin and gamithromycin) compared to oxytetracycline, no report of their use in practice has been identified
Lumpy skin disease (LSD)	- In Ethiopia, many feedlots buy bulls from Borena pastoral systems (on Borena markets), which have a history of LSD, without any knowledge of health status or veterinary inspection - Vaccination against LSD is part of national sanitary and phytosanitary requirements of animal quarantine for export markets; however, vaccination often starts too late to prevent transmission	- The cheap Neethling vaccine has been used mostly; however, vaccine failure has been reported and side effects occur - Heterologous vaccines are considered to induce less side effects, but are less effective	- Treatment of LSD is costly and does not always result in full recovery
Rift Valley Fever (RVF)	- Although mass vaccination is performed in outbreaks, ongoing mass vaccination is beyond the capacity of most SSA countries and vaccination is ceased in interepizootic periods	- The live attenuated Smithburn vaccine has the potential for reversion to virulence - Clone 13 is assumed to have the potential for genetic reassortment with virulent strains	- No specific treatment available
Bovine respiratory disease (BRD) complex	- A quarantine of newly purchased animals for 2–3 weeks is recommended to prevent the spread of BRD, but is not common practice in many areas and feedlots - Preconditioning of calves entering the feedlot significantly reduces morbidity, but is not common practice	- Efficacious vaccines are available, but uptake is low or nonexistent in parts of SSA	- Mainly “old” antimicrobials are used (e.g., long-acting tetracyclines, oxytetracyclines, tylosin, trimethoprim–sulfadiazine) - Reports on the common use of other antibiotics, such as new-generation macrolides, have not been identified through literature searches

## Data Availability

Not applicable.
